# Infiltration of inflammatory macrophages and neutrophils and widespread pyroptosis in lung drive influenza lethality in nonhuman primates

**DOI:** 10.1371/journal.ppat.1010395

**Published:** 2022-03-10

**Authors:** Jacqueline Corry, Gwenddolen Kettenburg, Amit A. Upadhyay, Megan Wallace, Michelle M. Marti, Elizabeth R. Wonderlich, Stephanie J. Bissel, Kyndal Goss, Timothy J. Sturgeon, Simon C. Watkins, Douglas S. Reed, Steven E. Bosinger, Simon M. Barratt-Boyes

**Affiliations:** 1 Department of Infectious Diseases & Microbiology, University of Pittsburgh, Pittsburgh, Pennsylvania, United States of America; 2 Yerkes NHP Genomics Core Laboratory, Yerkes National Primate Research Center, Emory University, Atlanta, Georgia, United States of America; 3 Division of Neuropathology, Department of Pathology, University of Pittsburgh, Pittsburgh, Pennsylvania, United States of America; 4 Center for Vaccine Research, University of Pittsburgh, Pittsburgh, Pennsylvania, United States of America; 5 Department of Cell Biology, University of Pittsburgh, Pittsburgh, Pennsylvania, United States of America; 6 Department of Immunology, University of Pittsburgh, Pittsburgh, Pennsylvania, United States of America; NYU School of Medicine, UNITED STATES

## Abstract

Severe influenza kills tens of thousands of individuals each year, yet the mechanisms driving lethality in humans are poorly understood. Here we used a unique translational model of lethal H5N1 influenza in cynomolgus macaques that utilizes inhalation of small-particle virus aerosols to define mechanisms driving lethal disease. RNA sequencing of lung tissue revealed an intense interferon response within two days of infection that resulted in widespread expression of interferon-stimulated genes, including inflammatory cytokines and chemokines. Macaques with lethal disease had rapid and profound loss of alveolar macrophages (AMs) and infiltration of activated CCR2^+^ CX3CR1^+^ interstitial macrophages (IMs) and neutrophils into lungs. Parallel changes of AMs and neutrophils in bronchoalveolar lavage (BAL) correlated with virus load when compared to macaques with mild influenza. Both AMs and IMs in lethal influenza were M1-type inflammatory macrophages which expressed neutrophil chemotactic factors, while neutrophils expressed genes associated with activation and generation of neutrophil extracellular traps (NETs). NETs were prominent in lung and were found in alveolar spaces as well as lung parenchyma. Genes associated with pyroptosis but not apoptosis were increased in lung, and activated inflammatory caspases, IL-1β and cleaved gasdermin D (GSDMD) were present in bronchoalveolar lavage fluid and lung homogenates. Cleaved GSDMD was expressed by lung macrophages and alveolar epithelial cells which were present in large numbers in alveolar spaces, consistent with loss of epithelial integrity. Cleaved GSDMD colocalized with viral NP-expressing cells in alveoli, reflecting pyroptosis of infected cells. These novel findings reveal that a potent interferon and inflammatory cascade in lung associated with infiltration of inflammatory macrophages and neutrophils, elaboration of NETs and cell death by pyroptosis mediates lethal H5N1 influenza in nonhuman primates, and by extension humans. These innate pathways represent promising therapeutic targets to prevent severe influenza and potentially other primary viral pneumonias in humans.

## Author summary

Influenza can cause acute lung injury and death, but the mechanisms resulting in lethal influenza in humans are not well understood. We used a novel model of lethal influenza in nonhuman primates caused by aerosol infection with highly pathogenic avian influenza virus that closely resembles human disease to define how the virus causes severe pneumonia. We found that a potent innate immune response starting with high-level production of interferons and inflammatory factors in the lung drives severe disease. Inflammatory cells including macrophages and neutrophils were recruited into lung because of this early response, which in turn led to release of neutrophil extracellular traps that blocked lung alveoli. In addition, a particularly inflammatory form of cell death known as pyroptosis occurred in lungs during lethal influenza. These new findings show that an intense interferon response leading to an inflammatory cascade of macrophages and neutrophils, release of neutrophil extracellular traps, and cell death by pyroptosis is responsible for acute lung injury in lethal influenza. These innate pathways could be targeted by drugs to prevent lung injury in critically ill influenza patients.

## Introduction

Human infection with highly pathogenic influenza viruses can produce primary viral pneumonia resulting in acute lung injury and death [1–4]. The mechanisms that contribute to lethal disease are poorly understood, but likely center around an over-exuberant innate immune response that produces hypercytokinemia, inflammation and cell death resulting in loss of alveolar epithelial barrier function [5]. Understanding the innate immune factors in the lung that cause inflammation and promote acute respiratory distress syndrome (ARDS) and lung injury in humans is central to developing therapies to prevent disease progression in critically ill and hospitalized influenza patients. Defining mechanisms of acute lung injury in severe influenza may also inform treatment of severe coronavirus disease 2019 (COVID-19), given the similarities between the two viral pneumonias [6].

There are several fundamental factors relating to the antiviral and innate responses to severe influenza in humans that remain ill-defined. Studies in nonhuman primates suggest that the severity of disease caused by infection with highly pathogenic H5N1 virus and the 1918 influenza virus is associated with attenuation of the antiviral interferon (IFN) response [7,8], and this is supported by *in vitro* studies [9]. In contrast, other reports indicate that a robust and sustained IFN response is a distinguishing feature of H5N1 infection in macaques [10]. In murine influenza models, type I and III IFN signaling is associated with impaired lung epithelial cell repair and increased disease severity [11]. Whether highly pathogenic influenza viruses promote or evade IFN responses *in vivo* and how this relates to pathogenesis is a central question that remains to be answered. Data on the innate cellular response to severe influenza are also incomplete. Alveolar macrophages (AMs) are an essential component of the innate response of the lung [12], and depletion of AMs markedly increases lethality of influenza in both pig and mouse models [13,14]. Recruitment of CCR2-expressing inflammatory macrophages and dendritic cells to lung is also considered a predominant cause of immune pathology and disease during influenza virus infection in murine models [15–17]. However, our understanding of macrophage biology in the context of severe influenza and ARDS in humans is limited [18]. Neutrophils can ameliorate lung injury during influenza [19], and activated neutrophils release neutrophil extracellular traps (NETs) that inactivate viruses and other pathogens, preventing spread [20]. However, NETs also promote thrombus formation [21,22] and can contribute to the pathogenesis of viral infections, including influenza [23–25]. Finally, current dogma points to a central role for apoptosis in influenza-induced cell death that disrupts the alveolar epithelial barrier leading to ARDS [26]. However, emerging studies indicate that inflammasome activation leading to caspase-driven pyroptosis is a critical factor in virus-induced lung injury [27].

To date it has been difficult to address these fundamental questions relating to influenza pathogenesis in humans as the field has lacked a suitable translational model of lethal influenza. Influenza virus infection in nonhuman primates generally produces only mild clinical disease even when using a strain that is highly lethal in humans such as avian influenza (H5N1), with some exceptions [8,10,28–31]. The conventional approach to H5N1 influenza virus inoculation in nonhuman primates uses virus inoculum in liquid suspension that is applied to oral, ocular, nasal and tracheal mucosal surfaces, but this is unlikely to deliver virus to cells in the alveoli which are the primary targets of infection. Based on the assumption that H5N1 influenza virus needs to reach the lower respiratory tract to effectively produce ARDS, we used inhalation of small-particle virus aerosols to develop a model of lethal influenza in cynomolgus macaques. The model is characterized by widespread virus replication in alveolar epithelial cells and AMs, massive production of inflammatory cytokines and chemokines in airways, and breakdown of the alveolar epithelial barrier leading to ARDS and death within days of virus exposure [32]. Using this powerful translational model, we analyzed the antiviral and innate immune responses to H5N1 influenza in the lung with the goal of identifying key pathologic pathways that drive lethal influenza disease in humans.

## Results

### Lethal H5N1 influenza activates potent interferon and inflammatory responses in lung

**Fig 1:**
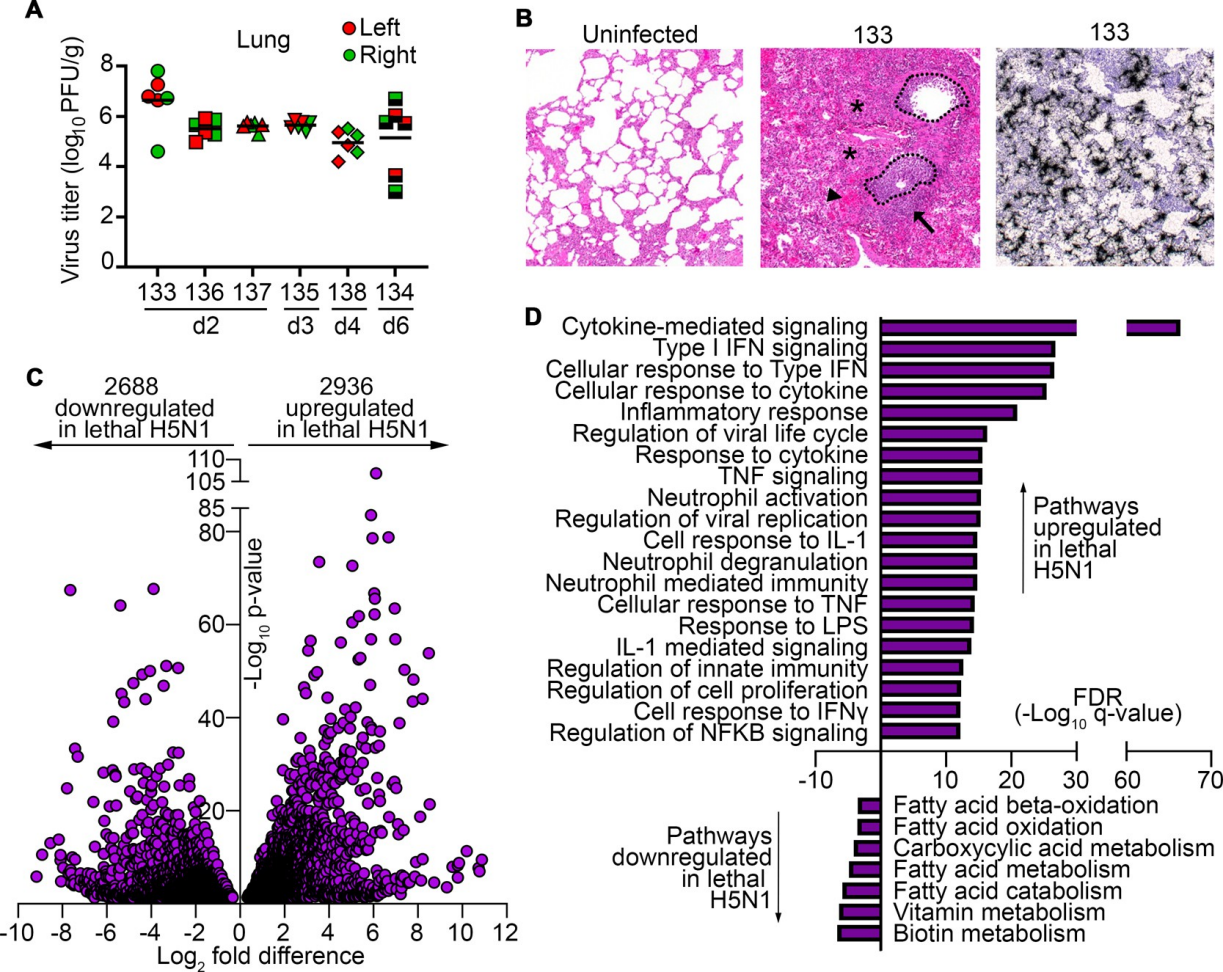
Lethal H5N1 influenza is associated with activation of antiviral and inflammatory pathways in lung. **(A)** Infectious virus titer in three different regions of left and right lung at necropsy from the six macaques. The day post infection of sacrifice is noted for each animal. **(B)** Hematoxylin and eosin staining of lungs from an uninfected macaque and infected macaque 133 at day 2 post infection, and *in situ* hybridization of influenza RNA in lung of macaque 133 at day 2 post infection. Dotted lines outline airways and highlight intense cellular infiltration in airways. Asterisks, arrowhead, and arrow mark alveolar consolidation, hemorrhage, and cellular infiltration in lung parenchyma, respectively. **(C)** Volcano plot of differentially expressed genes in lungs of uninfected and lethally infected macaques. Genes to the left of the Y-axis are decreased in lethal influenza relative to uninfected macaques, whereas genes to the right are increased in lethal influenza relative to uninfected macaques. **(D)** Bar graph showing enrichment false discovery rate (FDR) for the select biological pathways of differentially expressed genes in uninfected and infected lung. Pathways enriched in lethal influenza are shown above the X-axis, whereas pathways downregulated in lethal influenza are below X-axis. See also S1 and S2 Tables for a full list of up-and downregulated pathways.

We inoculated seven adult cynomolgus macaques with a mean dose of 6.72 log_10_ plaque forming units (PFU) of the highly pathogenic avian influenza virus human isolate A/Vietnam/1203/2004 (H5N1) by small-particle aerosol. All animals had severe influenza that progressed to ARDS and died or were humanely sacrificed between 2- and 6-days post infection, as detailed elsewhere [32]. Specimens from six of these macaques were available for additional study here. High titers of virus were isolated from three different regions of each lung at necropsy indicating diffuse replication across both lungs in all animals (Fig 1A). These findings were confirmed by semi-quantitative real-time PCR [32]. Histologically, lungs showed acute inflammation with widespread hemorrhage and infiltration of inflammatory cells as well as intra-alveolar exudate and consolidation. Viral RNA was readily detected in lung tissue by *in situ* hybridization (Fig 1B) [32].

To begin defining the host response to lethal influenza in this model, we performed bulk RNA sequencing of whole lung digests from the five infected macaques that died or were sacrificed early after infection, at day 2 (macaques 133, 136, 137), day 3 (135) and day 4 (138) (Fig 1A), along with three uninfected macaques and profiled transcriptional changes induced by lethal influenza using differential expression analysis. Similar numbers of genes were upregulated in the lungs of macaques with lethal influenza relative to uninfected macaques as were downregulated (2,936 genes upregulated versus 2,688 genes downregulated, respectively; Fig 1C). We then used Enrichr pathway analysis [33,34] and the GO Biological Processes library to identify significantly up- and downregulated pathways associated with these gene sets. Lethal influenza was uniformly associated with upregulation of inflammatory and antiviral pathways in the lung, including signaling through cytokines, type I IFN and IL-1, the inflammatory response and neutrophil activation (Fig 1D and S1 Table). Conversely, downregulated gene sets were less significantly changed and were primarily associated with fatty acid oxidation and metabolic pathways (Fig 1D and S2 Table).

**Fig 2:**
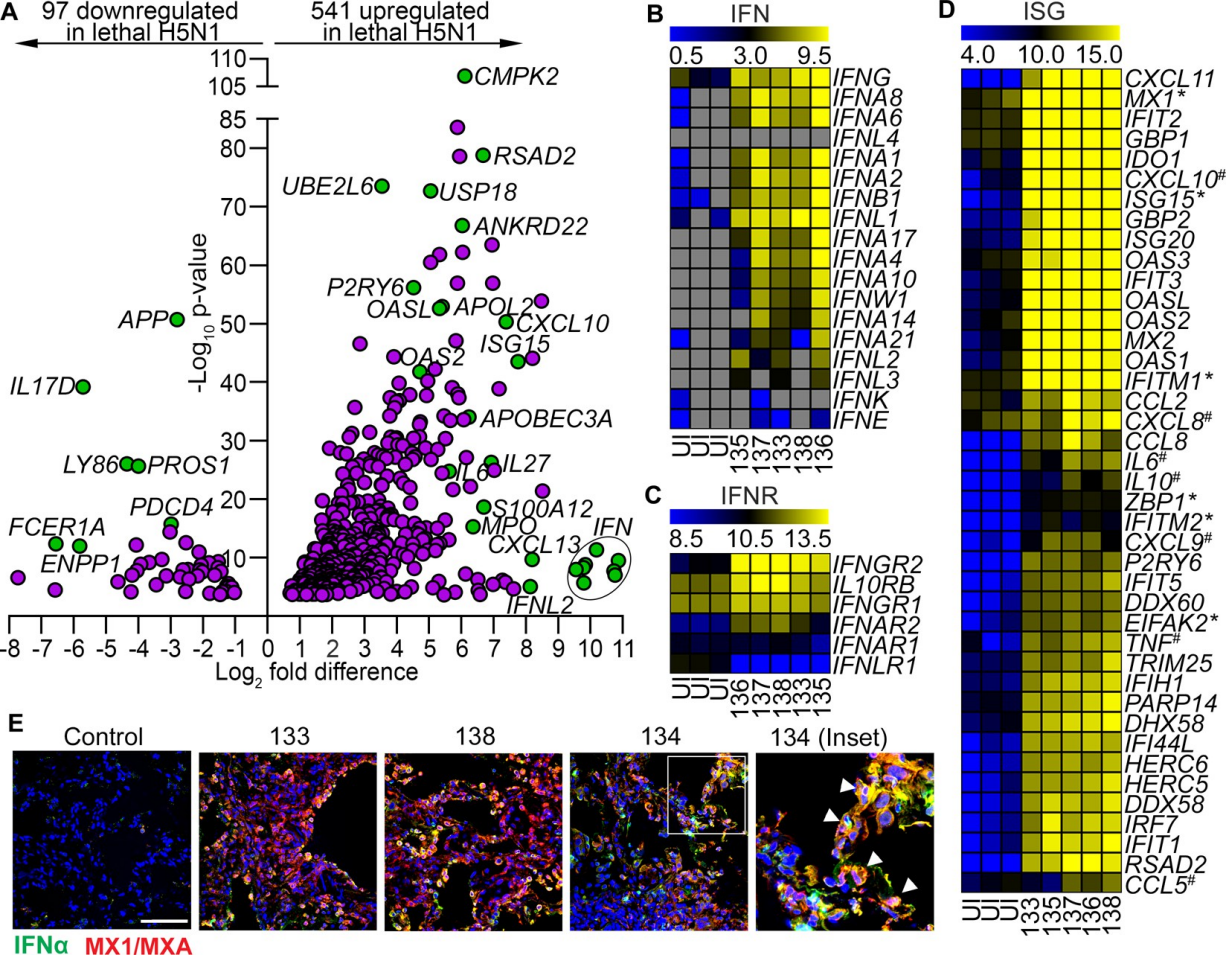
Expression of IFN and IFN-stimulated genes is highly upregulated in lung in lethal H5N1 influenza. **(A)** Volcano plot of IFN-stimulated genes (ISGs) and inflammatory genes that are differentially expressed in the lungs of uninfected and H5N1 infected macaques. Genes to the left of the Y-axis are decreased in lungs of macaques with lethal influenza relative to uninfected macaques, whereas genes to the right are increased in lethal influenza relative to uninfected macaques. Select genes are highlighted; circle denotes a concentration of *IFN* genes. See also S3 Table for a full list of genes. **(B-D)** Heat maps of log_2_ fragments per kilobase million (FPKM) values for IFN **(B)**, IFN receptors **(C)** and ISGs **(D)** in uninfected macaques (n = 3) and the identified macaques with lethal H5N1 influenza (n = 5). Grey boxes denote no expression. ISGs labeled with an asterisk (*) are known to restrict influenza replication. ISGs with a ^#^ have been shown to be high in the serum or plasma of patients hospitalized with severe H5N1 influenza. Additional ISGs are shown in S1 Fig. **(E)** Confocal images of lung sections from an uninfected macaque and the indicated infected macaques stained with antibodies to IFN-α2 (green) and MX1/MXA (red) and counterstained with DAPI nuclear dye. Double-labeled cells appear orange (arrowheads). Scale bar, 100 μm.

Because of the predominance of antiviral and inflammatory pathways associated with lethal influenza, we focused on genes related to these pathways in more detail. Of the 638 antiviral and inflammatory genes that were differentially expressed between lungs of uninfected macaques and macaques with lethal influenza, 541 (85%) were upregulated in response to infection, including inflammatory chemokines such as *CXCL10* and *CXCL13* and a cluster of IFN genes (Fig 2A and S3 Table). More in-depth heat map analyses showed that expression of genes encoding IFN-α, β, γ and λ as well as IFN receptors were uniformly increased in the lungs of animals with lethal influenza relative to uninfected controls (Fig 2B and 2C). There was some variation in expression of IFN genes across infected animals, but this was not associated with the different times post infection that tissues were harvested (Figs 1A and 2B). IFN-stimulated genes (ISG) were also broadly upregulated in lethal disease, including canonical ISGs and genes that have been shown to be important in restricting influenza infection, such as *MX1*, *ISG15* and *IFITM1* (Figs 2D and S1) [35–37]. To confirm our findings at the protein level, we performed immunohistochemistry on lung sections staining for IFN-α2 and MX1/MXA. Negligible expression of either protein was observed in lung from an uninfected macaque, as expected. Both IFN-α2 and MX1/MXA were widely expressed in lungs from macaques 133 (sacrificed at day 2 post infection), 138 (day 4) and 134 (day 6), indicating that the IFN and ISG responses were sustained at least for 6 days after virus inoculation. Notably, cells lining alveoli frequently co-expressed IFN-α2 and MX1/MXA to high levels (Fig 2E).

### Depletion of AMs and infiltration of M1-like inflammatory IMs in lethal H5N1 influenza

**Fig 3:**
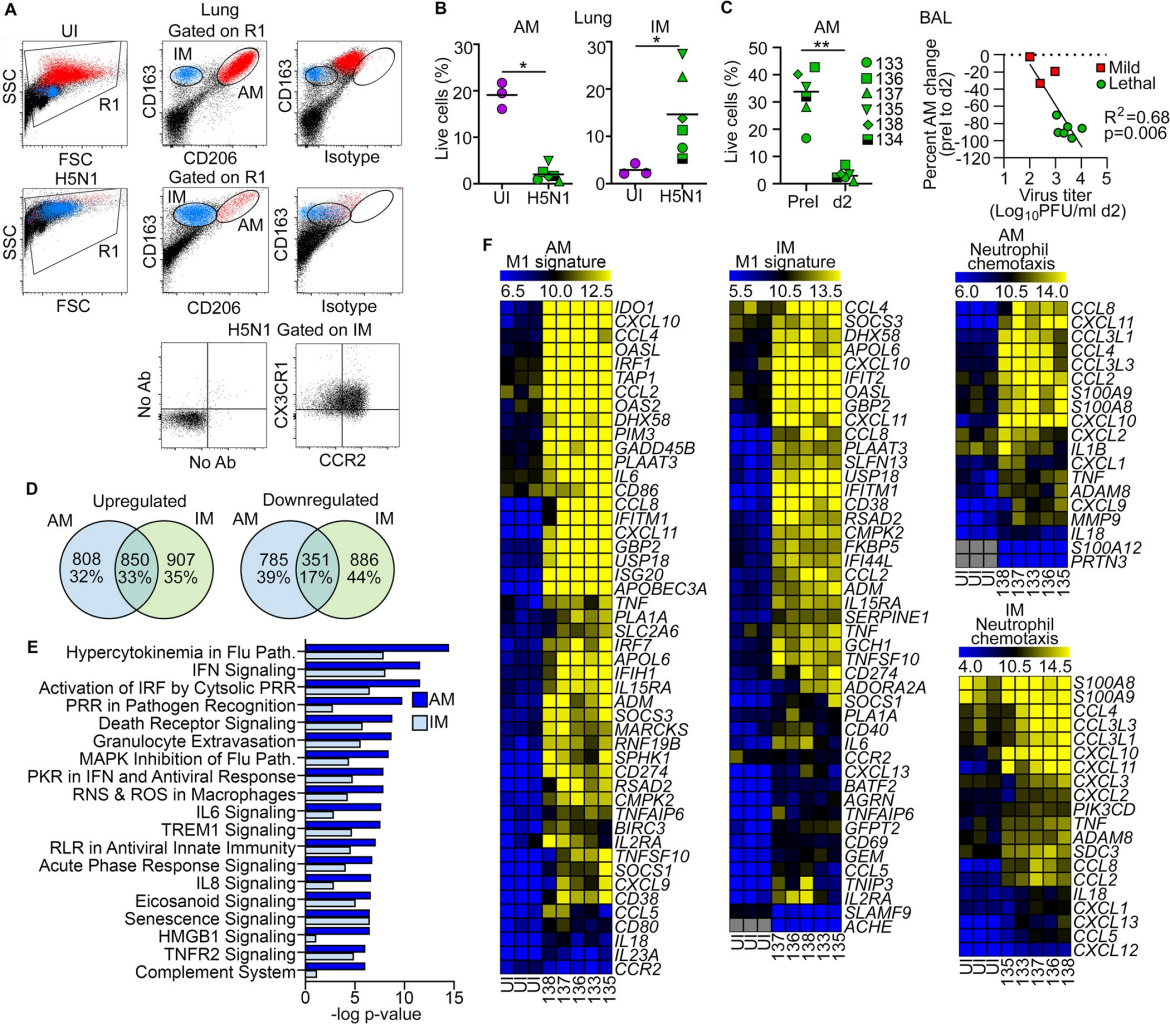
Lung macrophages are polarized toward an M1-like inflammatory state in lethal H5N1 influenza. **(A)** Flow cytometry dot plots showing representative analysis and gating of AMs (red) and IMs (blue) in lung of an uninfected macaque (UI) and a macaque with lethal H5N1 influenza stained with antibodies to CD163 and CD206. Cells from the infected macaque were additionally stained with antibodies to CCR2 and CX3CR1. Ovals are based on plots of CD163 and CD206 in uninfected and H5N1 infected animals and are overlaid onto the respective isotype control plots. FSC = forward scatter, SSC = side scatter. **(B)** Percent of AMs and IMs in lung homogenates at necropsy. (C) Left, Percent of AMs in BAL at pre-infection and day 2 post infection (*p<0,05, **p<0.01). Right, Linear regression demonstrating the relationship between virus titer in BAL at day 2 post infection and the change in AM frequency in BAL from pre-infection to 2 days post infection in macaques with mild (n = 3) and lethal influenza (n = 6). **(D)** Venn diagram of up- and downregulated genes expressed by AMs and IMs in lethal influenza relative to uninfected macaques. **(E)** Bar graph of pathways identified using Ingenuity Pathway Analysis as significantly differentially expressed in lethal influenza relative to uninfected macaques. See S4 and S5 Tables for a full list of canonical pathways. **(F)** Heat maps of differentially expressed genes in denoted pathways expressed as log_2_ FPKM values for M1-type gene signature and neutrophil chemotaxis in AMs and IMs in uninfected macaques (n = 3) and the identified macaques with lethal influenza (n = 5). See also S2 Fig for M2 genes and S6 and S7 Tables for list of M0, M1, and M2 genes. Grey boxes denote no expression.

We next determined how H5N1 influenza impacted the two major subsets of macrophages in the lung, the AMs and interstitial macrophages (IMs). Using flow cytometry, we gated viable lung cells based on forward scatter and side scatter and then stained for expression of CD163 and CD206 to define macrophages based on previous studies [32,38]. In lung suspensions from uninfected macaques, AMs were identified as highly autofluorescent cells that were CD163^++^ CD206^+^, whereas IMs were non-autofluorescent CD163^+^CD206^dim^ cells. The autofluorescence of AMs and the differential expression of CD206 were confirmed by inclusion of an isotype control in the place of CD206 antibody (Fig 3A). AM and IM subsets had a similar phenotype in infected macaque lung, but each subset was present in markedly different proportions as a function of infection. In macaques with lethal influenza, AMs in lung were reduced by 95% relative to uninfected controls, from an average of around 20% of all cells to just 2%. Conversely, IMs in the lung of uninfected animals represented only 3% of all cells but increased to an average of 15% with H5N1 influenza, although there was considerable variability between animals that did not associate with time to death (Fig 3B). Moreover, while IMs in uninfected macaques were relatively small and non-granular based on forward and side scatter characteristics compared to AMs, in lung suspensions from animals with lethal influenza the IMs were considerably larger and more granular, overlapping with the few remaining AMs, suggestive of activation (Fig 3A). In BAL there was a similarly profound reduction in AMs as a result of infection, from an average of 32% of live cells to 3% (Fig 3C). To determine if the loss of AMs in BAL was related to virus load, we analyzed AMs from an additional three cynomolgus macaques that were exposed to aerosolized influenza A/1203/2004 (H5N1) virus at approximately 1.5% the dose of lethal infection (4.9 log_10_ PFU) and presented with only mild influenza. We found a strong negative correlation between the percent change of AMs in BAL at 2 days post infection and the titer of virus in BAL fluid at the same time point when comparing mild and lethal influenza (Fig 3C). IMs were a negligible percentage of all BAL cells prior to infection, and this did not change after H5N1 influenza infection (S2 Fig). To determine the origin of the increased population of IMs in the lung of macaques with lethal influenza we stained lung cells for chemokine receptors known to be important in macrophage and monocyte recruitment [16,17]. The IMs in lethally infected macaques uniformly expressed CCR2 and CX3CR1 (Fig 3A), indicating they were likely recruited from the vasculature in response to infection [16,39].

We next analyzed the effect of H5N1 influenza on AMs and IMs by sorting the two subsets from the lungs of naïve (n = 3) and lethally infected animals (n = 5) and performing bulk RNA sequencing. AMs and IMs had differentially expressed genes that were either upregulated or downregulated in response to lethal infection, and between 32 and 44% of these genes were unique to each cell type, confirming that they were distinct macrophage populations (Fig 3D). We used Ingenuity Pathway Analysis [40] to better understand how these macrophage subsets responded to infection. Despite differential loss and recruitment, the predominant pathways that were upregulated in both AMs and IMs as a function of lethal infection were immune-related and/or inflammatory in nature, including hypercytokinemia, IFN signaling, and death receptor signaling (Fig 3E and S4 and S5 Tables). Heatmaps of genes associated with an M1 signature (S6 and S7 Tables) showed that both AMs and IMs had an overwhelming M1-like phenotype in response to lethal H5N1 influenza, consistent with inflammatory pathway activation (Fig 3F). In contrast, M2 signature genes (S6 and S7 Tables) did not distinguish between naïve and infected macaques (S3 Fig). Both AMs and IMs expressed chemokines that promote their continued recruitment to the lung, including *CCL2*, *CCL3*, *CXCL10* and *CXCL11*, in addition to marked upregulation of genes associated with neutrophil recruitment (Fig 3F).

### Lethal H5N1 influenza is associated with neutrophil recruitment and activation and NET release in lung

**Fig 4:**
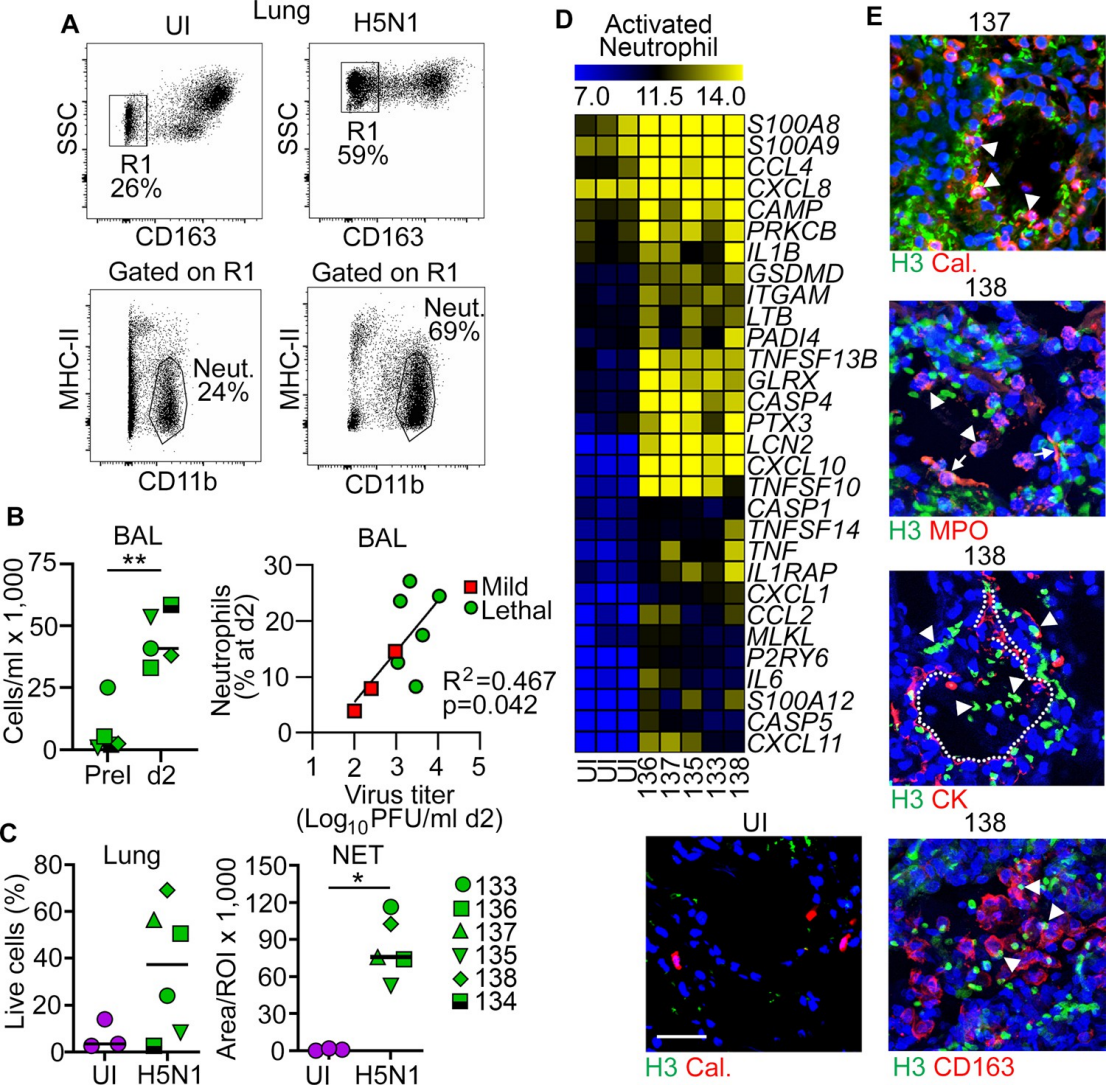
Influenza lethality is associated with neutrophil recruitment and activation and NET release in lung. **(A)** Gating strategy to identify and sort neutrophils (CD163^–^CD11b^+^MHC-II^–^) from whole lung suspensions of uninfected macaques and macaques with lethal H5N1 influenza by flow cytometry. **(B)** Left, Graphs showing the number of neutrophils in BAL at pre-infection and day 2 post infection. Right, Linear regression demonstrating the relationship between virus titer in BAL at day 2 post infection and the change in neutrophil frequency in BAL from pre-infection to 2 days post infection in macaques with mild (n = 3) and lethal influenza (n = 6). **(C)** The percentage of neutrophils in lung suspensions at necropsy, and the density of NETs in lung at necropsy for each macaque (*p<0,05, **p<0.01). **(D)** Heat maps of log_2_ FPKM values for neutrophil activation in sorted neutrophils from uninfected macaques (n = 3) and the identified macaques with lethal H5N1 influenza (n = 5). **(E)** Confocal images of lung sections from an uninfected macaque (UI) and the indicated macaques with lethal H5N1 influenza stained with antibodies to citrullinated histone H3 (green) and either calprotectin (Cal), myeloperoxidase (MPO), cytokeratin (CK) or CD163 (each in red) and counterstained with DAPI nuclear dye. Scale bar, 100 μm. Arrowheads in H3/Cal and H3/MPO indicate double-labeled cells (orange), arrows in H3/MPO indicate neutrophils actively extruding NETs. Arrowheads in H3/CK indicate NETs within alveolar spaces, dotted lines indicate outline of alveoli. Arrowheads in H3/CD163 show NETs in close proximity with macrophages.

We next analyzed neutrophils in BAL and lung suspensions by gating on CD163^–^MHC-II^–^CD11b^+^ cells (Fig 4A) [32]. Macaques with lethal H5N1 influenza had marked increases in the number of neutrophils in BAL at 2 days post infection relative to pre-infection. The increase in neutrophils in BAL was strongly correlated with virus load when macaques with lethal influenza were compared with the three macaques with mild influenza (Fig 4B). The percentage of neutrophils in the lung of macaques with lethal influenza was also increased relative to uninfected controls (Fig 4A and 4C). Neutrophils from the lung of uninfected macaques (n = 3) and macaques with lethal influenza (n = 5) were sorted and subjected to RNA sequencing to determine their response to infection in detail. Using the differentially expressed genes from pathways identified previously, we found that expression of genes associated with neutrophil activation were significantly increased in neutrophils from lungs of macaques with lethal influenza but not in uninfected macaques (Fig 4D). Neutrophils from infected macaque lungs expressed proinflammatory chemokines that also promote further neutrophil recruitment, including *CCL4*, *CXCL8* and *IL1B*. Sorted neutrophils had high expression of *CASP1*, *CASP4*, *PADI4* and *GSDMD*, genes that are required for NET production and release (NETosis). To visualize neutrophils and NETs in lung tissue directly, we stained sections of lung from uninfected macaques and macaques with lethal H5N1 influenza with antibodies to calprotectin and citrullinated H3 histone, respectively. There were abundant neutrophils in the lung of macaques with lethal influenza, and widespread staining with antibody to citrullinated histone H3, some of which co-labeled with calprotectin, both in the lung parenchyma and in airways (Fig 4E). Citrullinated histone H3 colocalized with myeloperoxidase within intact neutrophils and could be seen extending from neutrophils in the process of NET release (Fig 4E). NETs were readily identified within alveolar spaces, as highlighted by staining with antibody to cytokeratin to identify alveolar epithelial cells. In addition, NETs were in close approximation to CD163^+^ macrophages in the lung of infected macaques (Fig 4E). Quantification of citrullinated histone H3 staining revealed essentially no NETs in the lung of uninfected macaques but substantial area of NETs in lungs of macaques with lethal H5N1 influenza (Fig 4C).

### Pyroptosis is the primary mechanism of alveolar cell death in lethal H5N1 influenza

**Fig 5:**
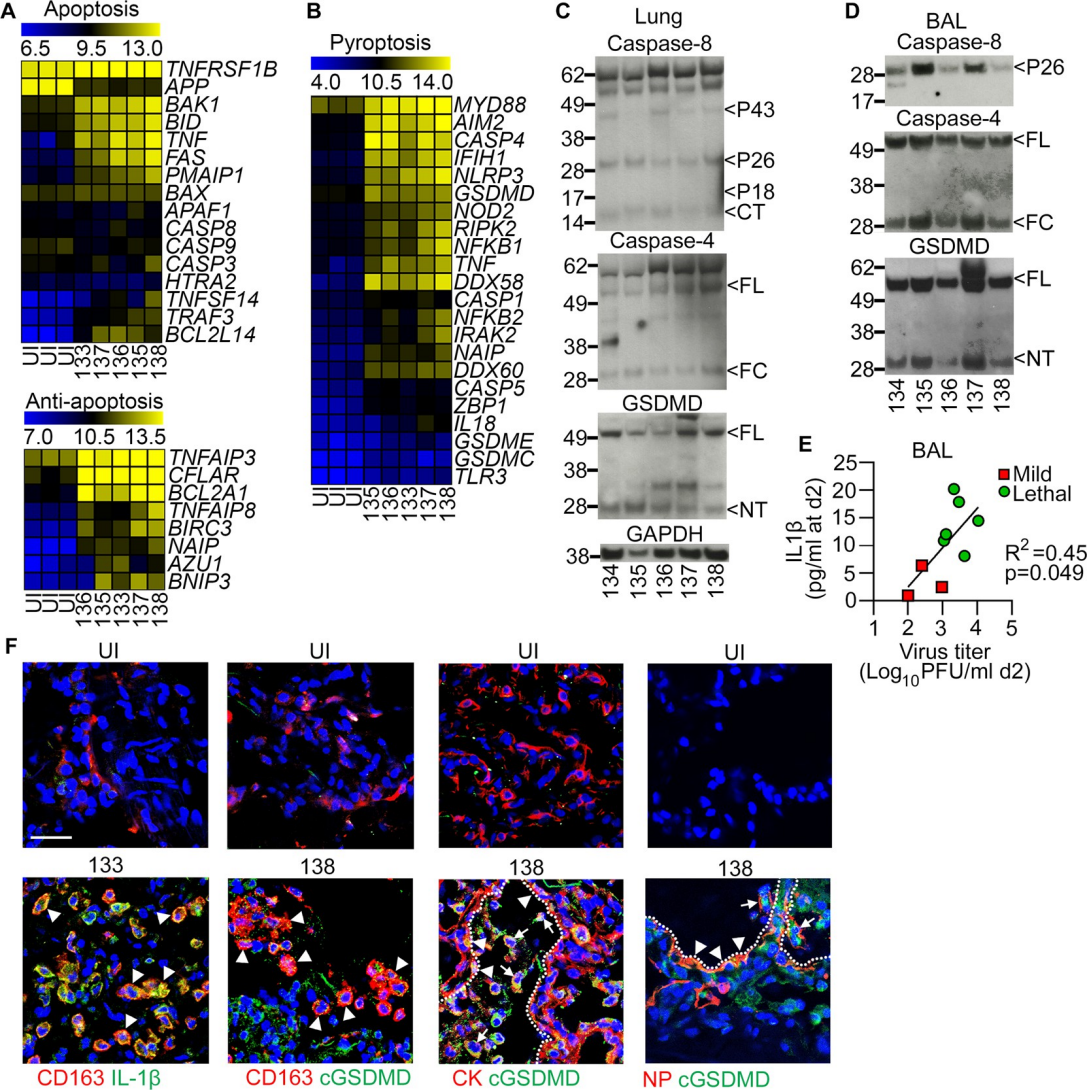
Pyroptosis is the primary mechanism of AM and alveolar epithelial cell death in lethal H5N1 influenza. **(A, B)** Heat maps of differentially expressed genes in lung lysates expressed as log_2_ FPKM values for proapoptotic and anti-apoptotic pathways (A) and pyroptotic cell death pathways (B) in uninfected macaques (n = 3) and the identified macaques with lethal influenza (n = 5). **(C, D)** Immunoblots of lung lysates **(C)** and BAL fluid **(D)** in identified macaques with lethal H5N1 influenza probed for caspase-8, caspase-4, GSDMD and GAPDH. Full-length and cleavage products are denoted with arrows. CT, c-terminal fragment; FL, full length; FC, fully cleaved; NT, n-terminal fragment. **(E)** Linear regression demonstrating the relationship between virus titer in BAL at day 2 post infection and the concentration of IL-1β in BAL fluid at 2 days post infection in macaques with mild (n = 3) and lethal influenza (n = 6). **(F)** Confocal images of lung sections from uninfected macaques and the indicated macaques with lethal H5N1 influenza stained with antibodies to CD163, cytokeratin (CK) or viral nucleoprotein (NP, each in red) and either IL-1β or cleaved GSDMD (cGSDMD, each in green) and counterstained with DAPI nuclear dye. Dotted lines show outline of alveoli. Arrowheads show double labeled cells (orange), arrows in CK/cGSDMD show cGSDMD-expressing alveolar epithelial cells that have been sloughed into alveolar lumen, arrows in NP/cGSDMD show infected cells expressing cGSDMD in alveolar lumen. Scale bars, 100 μm.

To determine the primary mechanism of cell death during lethal influenza in macaques, we first studied the expression of pro- and anti-apoptotic genes in lung tissue through RNA sequencing. The majority of proapoptotic genes were not differently expressed between uninfected macaques and macaques with lethal disease, and anti-apoptotic genes were expressed at greater levels in the lungs of macaques with severe influenza (Fig 5A). In contrast, genes associated with pyroptosis, including *CASP4*, *NLRP3* and *GSDMD*, were highly expressed in whole lung lysates from macaques with lethal influenza but not from uninfected animals (Fig 5B). To evaluate protein expression of key members of the apoptosis and pyroptosis pathways we did immunoblots of lung homogenates and BAL fluid from infected macaques. Expression of multiple fragments of caspase-8, an apoptosis-promoting caspase, was found in lung lysates of infected macaques, although the two key cleavage products P18 and P10 were not detected. Lung lysates expressed cleaved caspase-4, the active fragment of this inflammatory caspase, as well as full-length and the pore-forming N-terminal fragment of GSDMD that mediates pyroptotic cell death (Fig 5C). We next looked at protein expression in BAL fluid taken at 2 days post infection. The concentration of protein in BAL fluid was 10 to 24 times greater in macaques with lethal influenza (range 2.0–7.0 ug/ml) relative to uninfected macaques, consistent with protein exudate and leakage across airway epithelium in macaques with ARDS [32]. In BAL fluid from macaques with lethal influenza, only irrelevant caspase-8 cleavage byproducts were detected. In contrast, full-length and fully cleaved caspase-4, as well as full-length and cleaved GSDMD, were readily detected in BAL fluid from macaques with severe influenza (Fig 5D). The concentration of IL-1β, which is produced by the actions of inflammatory caspases and released from cells through pores generated by cleaved GSDMD, in BAL fluid at day 2 post infection was markedly increased in macaques with lethal influenza and was positively correlated with virus load when compared to macaques with mild influenza (Fig 5E).

Finally, we examined the expression of Il-1β and cleaved GSDMD in lung and its relationship to macrophages (CD163^+^) and alveolar epithelial cells (cytokeratin^+^) in uninfected macaques and those with lethal influenza by immunohistochemistry. Negligible staining for IL-1β or cleaved GSDMD was observed in lungs from uninfected macaques. There was widespread expression of IL-1β in lungs from macaques with severe influenza, and much of this expression was in macrophages, whose density was significantly increased with lethal disease relative to uninfected animals (Fig 5F). Severe influenza was also associated with increased expression of cleaved GSDMD, which colocalized with CD163 and with cytokeratin, reflecting pyroptosis of macrophages and alveolar epithelial cells respectively. Epithelial cells expressing cleaved GSDMD were present lining alveolar sacs but also within alveolar spaces, reflecting dead or dying epithelial cells that had been sloughed from alveoli (Fig 5F). Most GSDMD-expressing cells co-labeled with antibody to viral NP, indicating influenza virus infection, and these could be seen both in cells lining alveoli as well as cells within the alveolar lumen (Fig 5F). Together, these data show that lethal H5N1 influenza is associated with activation of inflammatory caspases, which leads to production and processing of IL-1β and GSDMD in lung macrophages and alveolar epithelial cells, consistent with pyroptosis.

## Discussion

Our findings in a translational model of lethal influenza in cynomolgus macaques shed light on the mechanisms of lethality caused by highly pathogenic avian influenza viruses, and by extension other severe primary viral pneumonias, in humans. We find that aerosolized H5N1 influenza virus infection induced a profound IFN response that promoted an inflammatory cascade in the lung. Lethal influenza was characterized by rapid depletion of AMs and infiltration of IMs with an overwhelming M1-like phenotype that further amplified the inflammatory response. Inflammatory macrophages released chemokines that induced recruitment of activated neutrophils, which elaborated NETs that occupied alveolar spaces. Infection induced activation of inflammatory caspases and production of pro-inflammatory IL-1β and cleaved GSDMD, leading to pyroptosis of alveolar epithelial cells and alveolar macrophages.

We found that a broad and intense IFN response in the lung is characteristic of severe H5N1 influenza. Macaques with ARDS caused by H5N1 influenza had uniform and high upregulation of type I, II and III IFNs, IFN receptors and ISG RNAs, as well as expression of IFN-α2 and MX1/MXA protein. Our prior work in these same macaques revealed that IFN-α2 protein concentration in the airways increased 2,000 times within just 2 days of lethal H5N1 infection, in association with virus titers of 10,000 PFU in airways [32]. This is not a function of lethal H5N1 influenza virus being resistant to IFN action [41] but rather we believe reflects the contribution of the IFN response to inflammation, which drives downstream effects that are detrimental to the lung. IFN responses also disrupt epithelial repair during the recovery phase of influenza in mouse models [11]. Single-cell RNA sequencing of PBMC from patients with mild or severe COVID-19 and severe influenza have highlighted the contribution of type I IFN-driven inflammatory responses in severity of both diseases [42]. A mouse model of SARS-CoV-2 infection has also shown an inflammatory role for type I IFN [43], similar to our findings in H5N1 influenza.

We characterized AM and IM populations based on differential expression of the scavenger receptor CD163 and the mannose receptor CD206, with AM being CD163^++^CD206^+^ and IM being CD163^+^CD206^dim^. In human lung tissue AMs and IMs have a similar phenotype and differential expression of CD206 as well as CD169, with AMs being CD11b^+^HLA-DR^++^CD206^++^CD169^+^ and IMs being CD11b^+^HLA-DR^++^CD206^+^CD169^–^ [44,45]. AMs in human lung tissue but not BAL have heterogeneity in CD163 expression, with both CD163^++^ and CD163^+^ subsets, an apparent distinction from macaques. Whether this reflects different ontogeny or function remains to be determined [44]. In macaques and humans BAL only contains the AM subset of macrophages; IM are absent from this compartment in healthy individuals [38,44], as we now confirm. In macaques with lethal H5N1 influenza, a central finding was the rapid depletion of AMs from both BAL and lung and the concurrent recruitment of IMs to lung. Both the few remaining AMs and the recruited IMs were activated, M1-type macrophages with high level expression of cytokine, chemokine and inflammatory genes all of which amplify inflammation and promote continued macrophage recruitment. M1-type IMs expressed CCR2, which mediates recruitment of inflammatory monocytes and DC from the circulation into lung [15–17], and CX3CR1, which mediates recruitment of CD16^+^ monocytes into tissues in humans [39]. Consistent with this, we have described a 10-fold increase in the number of CD14^+^CD16^+^ intermediate/inflammatory monocytes in blood within 2 days of H5N1 influenza infection in these same macaques [32]. These data reveal a pathologic role of inflammatory macrophages in severe influenza that is amplified by a positive feedback loop involving type I IFN. Similarly, proinflammatory M1-like macrophages that express high levels of the chemokines *CCL2*, *CCL3* and *CXCL10* are abundant in BAL fluid from patients with severe COVID-19, suggesting a pathologic role for inflammatory lung macrophages in COVID-19 as well [46].

The M1-type macrophages that predominate in the lung of macaques with lethal H5N1 influenza expressed high levels of genes involved in neutrophil recruitment, and as a result the number of neutrophils in BAL and lung increased markedly. Recruited neutrophils were activated and inflammatory and expressed high levels of cytokines and chemokines that promote their continued recruitment, consistent with a feedforward inflammatory circuit driving the pathogenesis of lethal influenza [47,48]. Activated neutrophils released NETs which were identified within alveolar spaces, indicating a contribution to airway obstruction and inhibition of gas exchange. Similarly, obstruction of airways by NETs is seen in severe respiratory syncytial virus infection [49]. While our in-situ analysis of fixed lung tissue did not allow us to distinguish between vital and suicidal NETosis, the co-expression of citrullinated H3 histone and myeloperoxidase in apparently intact neutrophils suggests that at least a component of NETs may have been produced from viable neutrophils [50,51]. NETs also facilitate acute lung injury by promoting the polarization of M1-type inflammatory macrophages [52], and inducing thrombosis [22,53,54]. NETs have been shown to induce plasmacytoid dendritic cells to produce type I IFN [55,56], which would exacerbate the IFN response already induced by H5N1 influenza. NETs are induced by inflammatory caspases in murine models of influenza, and their disruption by DNase reduces influenza severity, consistent with a pathologic role [57]. In humans, neutrophil activation and NETosis in blood correlate with influenza disease severity [24,58]. These findings show that recruitment of activated neutrophils and release of NETs into lung airways and parenchyma likely contribute to pathology in severe influenza. Similarly, studies of SARS-CoV-2 infection in rhesus macaques reveal that inflammatory lung macrophages secrete chemokines that recruit neutrophils and promote NETosis, a pathologic pathway that can be blocked by the JAK1/JAK2 inhibitor baricitinib [59]. It is interesting to note that activation of eosinophils has been linked to lung hyperinflammation in patients hospitalized with COVID-19 [60,61], suggesting that polymorphonuclear granulocytes in general may be involved in the pathogenesis of severe viral pneumonias.

Approximately 20 and 30% of alveolar epithelial cells and AMs, respectively, are infected in macaques following inhalation of high-titer aerosolized H5N1 influenza [32], and our current findings reveal that these cells die by pyroptosis. Influenza virus activates inflammatory caspases when they are recruited to NLRP3 inflammasomes [62–64] and this in turn produces cleavage of the N-terminal fragment of GSDMD that is essential for IL-1β release and cell lysis during pyroptosis [65,66]. Cell death by pyroptosis is also promoted by the IFN response [67], suggesting a further role for IFNs in influenza pathogenesis. Cleaved GSDMD colocalized with macrophages and alveolar epithelial cells and with viral NP protein in lung sections of macaques with lethal influenza, consistent with pyroptosis of infected cells. In the lung and BAL fluid there were high levels of IL-1β, and a large proportion of IL-1β-secreting cells were CD163^+^ macrophages. NETs also promote caspase-dependent pyroptosis of macrophages [54], indicating that both virus-infected macrophages as well as macrophages in intimate contact with NETs undergo pyroptosis. Death of infected alveolar epithelial cells by pyroptosis would result in loss of the alveolar epithelial barrier leading to pulmonary edema and fluid accumulation in airways, driving ARDS [32]. Our data do not support a role for apoptosis in cell death in severe H5N1 influenza virus infection. Prior studies documenting apoptosis in lung autopsy specimens from individuals succumbing to H5N1 influenza used the terminal deoxynucleotidyl transferase-mediated dUTP-biotin nick end-labeling (TUNEL) assay [68], however TUNEL staining does not differentiate between apoptosis and pyroptosis [69].

The identification of pyroptosis as a key pathologic effector in severe influenza opens new opportunities for therapy to prevent development of ARDS in critically ill and hospitalized patients. Targeting GSDMD is particularly appealing [70], given its major role in IL-1β release and both pyroptosis and NETosis. One promising approach is to use the FDA-approved drug disulfiram, which inhibits pyroptosis by blocking the pore-forming function of GSDMD [71]. Inhibitors of peptidyl arginine deiminase 4 that mediates citrullination of histones such as BB-Cl-amidine have shown promise in blocking NET formation and inflammation in experimental models of lupus and arthritis [72,73]. Recent data also point to a critical contribution of pyroptosis in the severity of COVID-19, based on increased serum levels of lactate dehydrogenase, a cytosolic enzyme that is released from ruptured cells because of pyroptosis [74]. Collectively, these findings indicate that inflammasome-mediate caspase activation and pyroptosis are likely to be good therapeutic targets in the treatment of patients hospitalized with severe or critical COVID-19 [75].

## Materials and methods

### Ethics statement

Experiments with nonhuman primates were conducted in accordance with the National Institutes of Health recommendations in the *Guide for the Care and Use of Laboratory Animals*. The following protocols were approved and overseen by the Institutional Animal Care and Use Committee (protocol # 15055917, 18073153) and the Institutional Biosafety Committee (protocol # 055–15) at the University of Pittsburgh. Macaques were observed twice daily and were given a combined clinical score based on body temperature, clinical appearance and respiratory signs at each observation. Samples were collected following sedation with ketamine hydrochloride, and virus infection was carried out under anesthesia with Telazol. If an animal had a combined score that reflected advancing and irreversible disease the macaque was humanely euthanized to ameliorate suffering. Euthanasia was achieved using pentobarbital sodium and phenytoin sodium injection followed by cardiac perfusion.

### Oversight

Animal and biosafety level 3 (BSL-3) experiments were performed in the University of Pittsburgh Regional Biocontainment Laboratory, which is a registered entity with both the Center for Disease Control and Prevention as well as the U.S. Department of Agriculture for work with highly pathogenic avian influenza viruses. All personnel were required to wear appropriate personal protective equipment including powered air-purifying respirators (3M Versaflo) when working with infectious virus and infected macaques.

### Animals and virus

Tissues from six adult cynomolgus macaques (*Macaca fascicularis*) of Mauritius origin from a group of seven that were infected by small-particle aerosol with an average of 6.72 log_10_ PFUs of highly pathogenic avian influenza virus A/Vietnam/1203/2004 (H5N1) strain and sacrificed or died between 2 and 6 days post infection due to ARDS [32] were used in the study. BAL samples taken at day 2 post infection from an additional three macaques that were infected in the same manner with approximately 4.9 log10 PFUs of the same virus stock and presented with mild influenza were also used. Tissues from three uninfected macaques were used as controls. Details of aerosol exposure, virus quantification and tissue processing are previously described [32]. Virus quantification in BAL and snap frozen lung specimens by plaque assay was done as previously described [32].

### In situ *hybridization and immunofluorescence*

In situ hybridization of formalin-fixed tissues and immunohistochemistry of paraformaldehyde-fixed tissues were performed as described [32]. The primary antibodies used for immunohistochemistry were against calprotectin (MAC 387; ThermoFisher Scientific), CD163 (GHI/61; Invitrogen), cytokeratin (AE1/AE3; Abcam), cleaved GSDMD (EPR20829-408; Abcam), histone H3 (ab18521; Abcam), IFN-1α (MMHA-2; Fisher), IL-1β (6E10; Novus Biologicals), MxA/Mx1 (4812; Novus Biologicals) and influenza A nucleoprotein (DPJY03, BEI Resources). To quantify expression in tissue, TIFF images taken by confocal microscopy were analyzed using Nikon NIS Elements version 4.50.00. Regions of interest were drawn to encapsulate lung tissue and exclude edges of tissue/airways in the tissue where no tissue is present. Microscope slides were imaged non-repetitively for 10 images per tissue section. Stains of interest were then quantified by recording the binary area (recorded as pixels) and averaging the values to generate individual data points per animal, which were averaged for presentation as single data points.

### Flow cytometry and cell sorting

Flow cytometry and antibody staining of cells in suspension were done as previously described [32], with the addition of antibodies to CCR2 (REA264; Miltenyi) and CX3CR1 (2A9-1; Thermo Fisher). To sort cells from lung suspensions, previously frozen lung cell suspensions were labeled with antibodies to CD163, CD206, CD11b and MHC class II and sorted under BSL-3 containment using a BD FACSAria cell sorter. Cells were gated on CD163^++^CD206^+^ for AMs, CD163^+^CD206^dim^ for IMs, and CD163^–^CD11b^+^MHC-II^−^for neutrophils. Analysis during sorting indicated greater than 90% purity of each cell population. Cells were collected directly into Buffer RLT (Qiagen) with β-mercaptoethanol and cell lysates stored at -80°C. Replicates of cell lysates were shown to be free of infectious virus by plaque assay prior to removal from the containment facility.

### RNA Sequencing

RNA extraction and NGS library preparation was performed in the Yerkes NHP Genomics Core. RNA was isolated using RNeasy Micro Kits (Qiagen) with on-column DNase Digestion (Qiagen). RNA quality and quantity were determined using a Bioanalyzer RNA Pico chip (Agilent). RNA was converted to cDNA and amplified using the Clontech SMART-Seq v4 Ultra Low Input RNA kit (Takara Bio) according to the manufacturer’s instructions. Amplified cDNA was fragmented and appended with dual-indexed bar codes using the NexteraXT DNA Library Preparation kit (Illumina). Libraries were validated by capillary electrophoresis on an Agilent 4200 TapeStation, pooled at equimolar concentrations, and sequenced on an Illumina HiSeq3000 at 100SR, yielding 20–25 million reads per sample.

### Bioinformatics and data analysis

The quality of sequenced reads was assessed with FastQC. The Spliced Transcript Alignment to a Reference (STAR) [72] aligner version 2.5.2b was used to align raw reads to build version 7.8.2 of the MacaM genome reference, kindly provided by Dr. R Norgren, University of Nebraska. Transcript abundance estimates were calculated internal to the STAR aligner using the algorithm of htseq-count [74]. DESeq2 was used for normalization [75], producing both a normalized read count table and a regularized log expression table, and to calculate counts for basic annotated genes. Volcano plots, bar graphs, and x,y graphs were generated using GraphPad Prism software. Differentially expressed genes were used with either the q- or p- value with a cut off of 0.05 or 0.01 as stated. Up- or downregulated genes (p<0.01 log2 fold change <-1 or >1) were separately input into the Enrichr site for the lung enrichment data (Figs 1D and S1 and S2 Tables) with q<0.05 considered to be significant. Hierarchical clustering was performed and heatmaps generated using MeV software using the log_2_ FPKM values, cluster maps removed for ease and space.

### Immunoblot analysis

Protein assay was performed using the Pierce Rapid Gold BCA Protein Assay kit (Thermo Fisher Scientific) on snap frozen lung lysate or BAL fluid. 25 ug of lysate or equal volumes of BAL fluid (to add 25 ug of the most concentrated sample) were prepared for each of the antibodies to be used in Bolt LDS sample buffer and Bolt sample reducing agent (Thermo Fisher Scientific) and denatured at 70°C for 10 minutes. Samples and SeeBlue Plus2 pre-stained protein ladder were run on Bolt Bis-Tris 4–12% gel (Thermo Fisher Scientific) and transferred to nitrocellulose. Blots were rinsed with water, imaged and de-stained with 1X tris buffered saline with 0.05% Tween 20 (TBS-T). Blots were then blocked with 5% milk and incubated with primary antibody followed by secondary antibody. Immunoblots were exposed to Super Signal West Pico plus or Fempto chemiluminescent substrate (Thermo Fisher Scientific) and exposed to Amersham HyperFilm ECL (Fisher) film for 1 second to 5 minutes and developed using SRX-101A Medical Film Processor (Konica Minolta). The following primary antibodies were used: caspase-4 (Clone: 4B9; Santa Cruz), caspase-8 (90A992; Novus Biologicals); and GSDMDC1 (64-Y; Santa Cruz). Separate gels were run for each of the antibodies using the same sample preparation on the same day with the same loading control.

### Statistical analysis

All statistical analyses were performed in GraphPad Prism v8.0–9.0 (GraphPad Software). A *p* value of <0.05 was considered significant. Comparisons between groups were two-tailed t-tests with Welch’s corrections. Comparisons of multiple groups was performed with a Kruskal-Wallis ANOVA, and if the null was rejected, further analysis was performed between groups using Dunn’s multiple comparisons test.

## Supporting information

S1 Fig Expression of interferon-stimulated genes are increased in the lung of macaques with severe influenza. Heat maps of differentially expressed ISGs in lungs from uninfected macaques (n = 3) and macaques with lethal influenza (severe, n = 5). The panel of ISGs is based on a previous publication [35]. Heat maps are expressed as log_2_ FPKM values. Genes are separated by expression value. Grey boxes denote no expression.
(TIF)

S2 Fig Interstitial macrophages are rare in BAL both before and after H5N1 influenza virus infection. Percent of total cells in bronchoalveolar lavage that is IM at pre-infection and 2 days post infection in animals with lethal influenza disease.
(TIF)

S3 Fig Pulmonary macrophages in severe influenza do not have an M2-like macrophage signature. Heat maps of differentially expressed genes previously identified as M2-macrophage genes in sorted AM and IM from uninfected macaques (n = 3) and macaques with lethal influenza (severe, n = 5). Genes expressed as log_2_ FPKM values. Grey boxes denote no expression.
(TIF)

S1 Table Enrichr Pathway analysis. Pathways and associated genes shown in Fig 1 that are upregulated in H5N1 influenza.
(XLSX)

S2 Table Enrichr Pathway analysis. Pathways and associated genes shown in Fig 1 that are downregulated in H5N1 influenza.
(XLSX)

S3 Table Interferon, inflammatory and antiviral genes. Genes used to generate the volcano plot of Fig 2A.
(XLSX)

S4 Table Ingenuity Pathway Analysis. Canonical pathways and associated genes for AMs shown in Fig 3E.
(XLSX)

S5 Table Ingenuity Pathway Analysis. Canonical pathways and associated genes for IMs shown in Fig 3E.
(XLSX)

S6 Table M0, M1 and M2-type genes. Genes expressed in AMs used in Figs 3F and S3.
(XLSX)

S7 Table M0, M1 and M2-type genes. Genes expressed in IMs used in Figs 3F and S3.
(XLSX)
