# Supplementary figures and images for: Infiltration of inflammatory macrophages and neutrophils and widespread pyroptosis in lung drive influenza lethality in nonhuman primates
Source: PLoS Pathog. 2022 Mar 10;18(3):e1010395. doi: 10.1371/journal.ppat.1010395 (PMC8939778; doi:10.1371/journal.ppat.1010395)

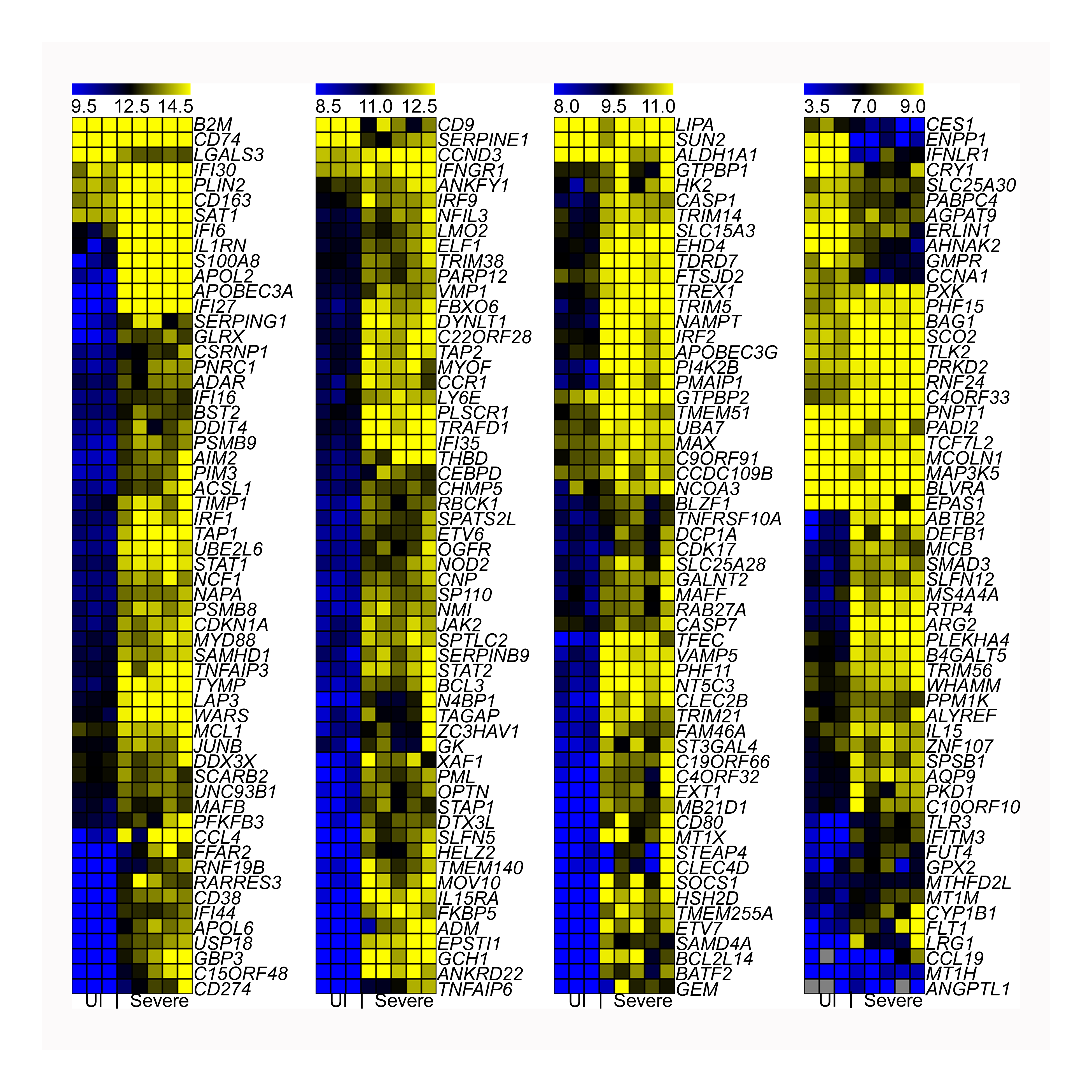

Supplement: S1 Fig — Heat maps of differentially expressed ISGs in lungs from uninfected macaques (n = 3) and macaques with lethal influenza (severe, n = 5). The panel of ISGs is based on a previous publication [35]. Heat maps are expressed as log2 FPKM values. Genes are separated by expression value. Grey boxes denote no expression. (TIF) [file ppat.1010395.s001.tif]

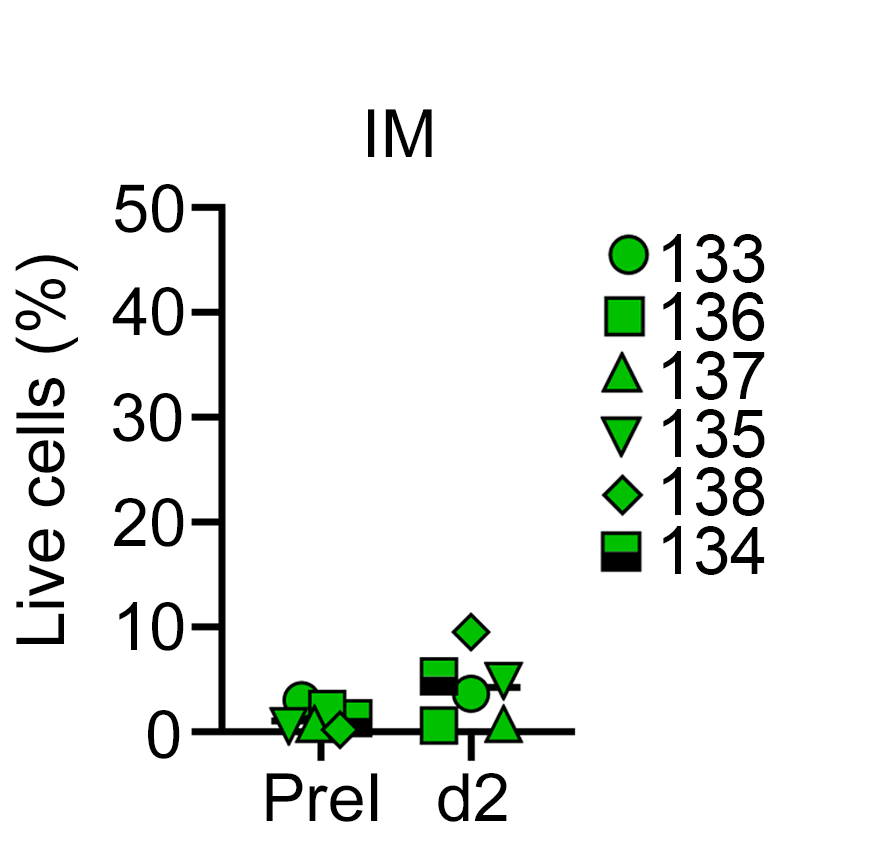

Supplement: S2 Fig — Percent of total cells in bronchoalveolar lavage that is IM at pre-infection and 2 days post infection in animals with lethal influenza disease. (TIF) [file ppat.1010395.s002.tif]

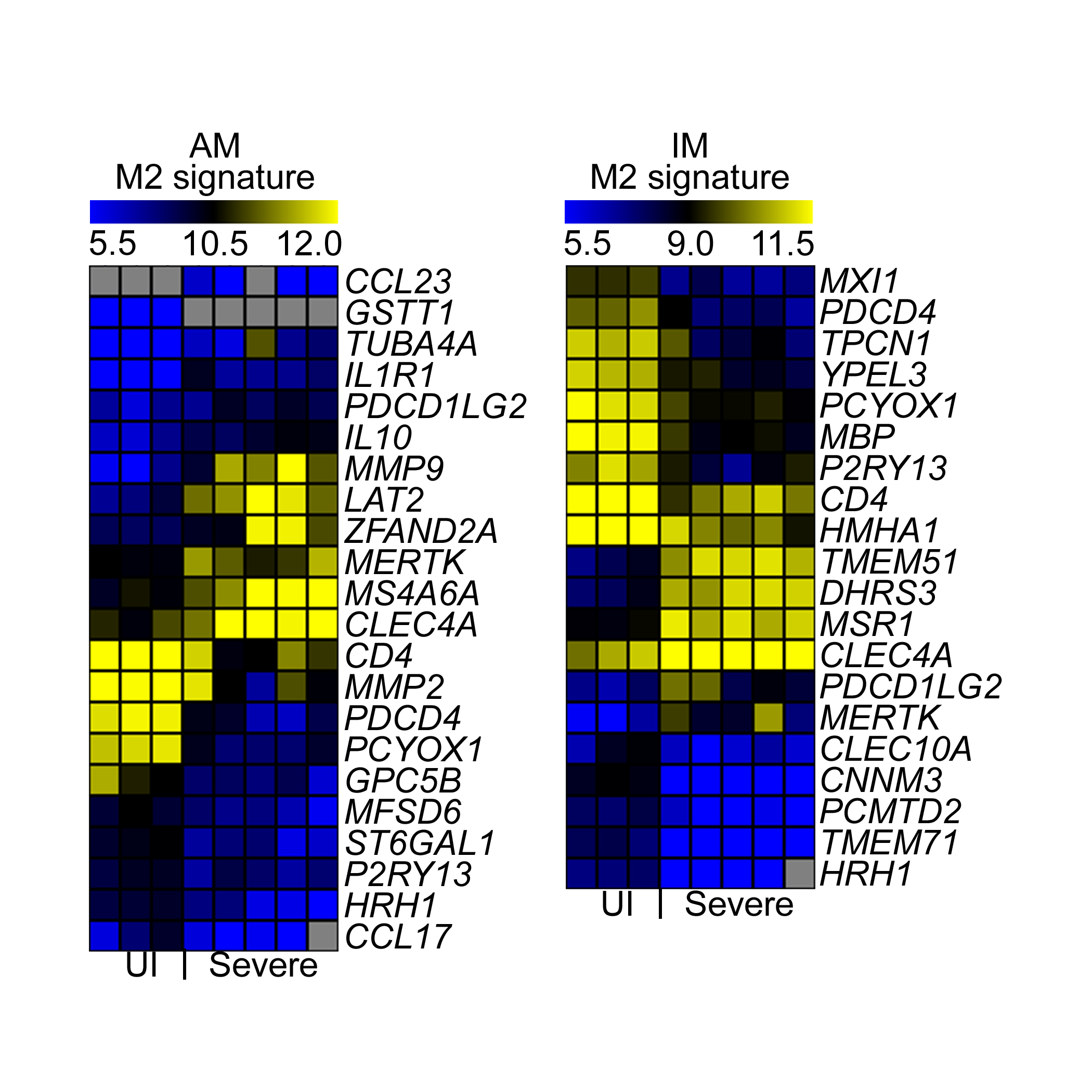

Supplement: S3 Fig — Heat maps of differentially expressed genes previously identified as M2-macrophage genes in sorted AM and IM from uninfected macaques (n = 3) and macaques with lethal influenza (severe, n = 5). Genes expressed as log2 FPKM values. Grey boxes denote no expression. (TIF) [file ppat.1010395.s003.tif]
